# Treatment of Acute Broncho-Pneumonia in Children*Read before the Twentieth Semi-Annual Meeting of the Southern California Medical Society, held in Los Angeles December 1 and 2, 1897.

**Published:** 1898-03

**Authors:** J. H. Seymour

**Affiliations:** Los Angeles, Cal.


### TREATMENT OF ACUTE BRONCHO-PNEUMONIA IN CHILDREN.*

BY J. H. SEYMOUR, M. D., LOS ANGELES, CAL.

Broncho-pneumonia is a disease very frequently met with, and is one of the most serious of the acute pulmonary affections of early childhood; consequently, the subject of treatment is one of great importance to the general practitioner. The condition present and to be met is one of local inflammation which has spread from above downward, from the larger into the terminal bronchi and adjacent alveoli—the alveoli are never the first to become involved. A careful inquiry will elicit the fact of a pre-existing bronchial inflammation. With a thorough appreciation that broncho-pneumonia is a secondary disease, it becomes evident that something can be done in the way of prophylaxis. I believe it to be largely a preventable disease. Rational prophylaxis, however, should commence at birth, and consists in fulfilling in the highest possible degree the laws of hygiene and diet. Improperly nourished, rachitic and scrofulous children (so many examples of whom we see among those who have been fed on some one of the many proprietary foods on the market, nearly all of which are deficient in one or more of the elements necessary for the building up of tissue capable of resisting disease encroachments), these little unfortunates, I say, with pale and delicate skins and still more sensitive mucous membranes are particularly prone to repeated attacks of catarrhal inflammations, involving not only the nasal and pharyngeal mucous membranes but frequently by extension the bronchial and pulmonic.

We can accomplish much by attention to this question of diet for the young infant requiring an artificial rearing.

Likewise of importance is the question of the proper clothing to be worn by young children. Extremes in this matter are to be avoided, the "coddling" of children is to be deprecated equally with that of over exposure. Loose-fitting flannel undergarments of medium weight, covering the arms and legs, should be worn during the winter months, at least, in this climate. The child should be given each morning a cold or tepid bath followed by a brisk rubbing, particularly of the chest and throat. Pure air and out-of-door exercise should be granted them, the former in unlimited quantity, the latter regularly, whenever the weather permits.

---

*Read before the Twentieth Semi-Annual Meeting of the Southern California Medical Society, held in Los Angeles December 1 and 2, 1897.

On the appearance of catarrhal symptoms, however, the child should be confined to the house, the rooms kept at an equable temperature and a mild laxative given, followed by a little paregoric or Dover's powder. When convinced that the inflammatory action has extended from the larger bronchial tubes into the terminal bronchi and alveoli, a fact made known by no sharp line of demarcation, but rather by an intensifying of all the symptoms, temperature, pulse, respiration, etc., and particularly by the change in the character of the cough and cry; when, I say, we are convinced that we have catarrhal pneumonia to deal with, our line of treatment will be an expectant one, directed towards supporting the vital forces and meeting the indications as they may arise.

The strength of the little patient should be conserved in every possible manner, for this disease makes rapid inroads upon the vitality of infants and young children. Careful nursing occupies first place in the successful treatment of broncho-pneumonia. The child should be put to bed, not held in the arms or toted about the house. The room should be a sunny one and capable of thorough ventilation, that with an open fireplace being particularly desirable. The temperature of the room should be kept equable—from 68° to 70°F.—and the atmosphere rendered moist by means of a bronchitis-kettle. The inhalation of steam is of the greatest service in these cases; cough, frequently very annoying, is lessened, the viscid secretions are rendered more fluid and expectoration facilitated and in so far favors a limitation of the pneumonic process, bearing in mind that the condition present is to a certain extent a mechanical one, that the embarrassed breathing and labored heart's action are caused by the presence of tenacious mucus and obstructed bronchi with the resulting atelectasis. The importance of keeping these secretions in a liquid condition can not be overestimated, and in steam we have our best agent for accomplishing this result.

The child should be made as comfortable as possible and its position in bed frequently changed in order to lessen any tendency there may be to hypostatic congestion. A loose flannel night-dress should be worn, large enough to enable us to reach the chest with our topical remedies without too great annoyance to the patient.

With reference to external applications, what is it best to do? The classical flaxseed-meal poultice, continuously, applied, I have long since discarded as a routine measure. Occasionally for the relief of pain, and when other means fail, resort will be had for a few hours to this. Continuously used however, they do more harm than good. The difficulty of having them properly applied, the worry and annoyance which their frequent application gives the child, the danger, through neglect, of their becoming cold and soggy, but above all, the weighting of the chest with a heavy poultice, supplies additional work for the little sufferer whose every effort is being put forth to supply the needed amount of oxygen (through the uncrippled portions of lung tissue) to a blood current already overcharged with carbon dioxide.

It is to the maintaining of the respiratory function that all our efforts are being directed, for in these cases death, when it comes, results from failure of the respiration, not from heart failure. I would, therefore, unreservedly condemn the continuous use of flax-seed poultices in broncho-pneumonia. By the judicious use of rubefacients, however, we can materially relieve the congestion of the pulmonary and bronchial circulation, and one of the best of these to use is the old time mustard-paste, of the strength of one part of mustard to four of flour. They should be left on until the skin is moderately reddened and reapplied several times a day throughout the disease. Camphorated oil liberally used and thoroughly rubbed onto the chest makes a satisfactory counter-irritant in mild cases.

The chest should, throughout the entire course of the disease, be encased in a loose-fitting cotton jacket. This can easily and quickly be made out of cotton batting and cheese-cloth, or flannel.

The general indications to be met in the treatment of catarrhal pneumonia are three in number: 1. The support of the patient's vital forces. 2. The controlling of the pyrexia. 3. The assisting (by every means direct or indirect) to maintain the respiratory function until resolution takes place. The inflammation can not be checked by any abortive line of treatment. The best results are obtained by promptly meeting the indications as they may arise.

In the beginning of the attack a thorough unloading of the alimentary canal, by means of calomel and bicarbonate of sodium in small doses repeated hourly until free action is obtained, is always beneficial; vomiting and nausea, often present, are checked and the stomach and bowels rendered more tolerant of food.

In the matter of feeding, care should be exercised not to become overzealous in pushing the nourishment. Gastro-intestinal irritation, with the attendant accumulation of flatus, renders respiration still more difficult, and its prevention is one of the most important duties of the physician. Easily digested nourishment, frequently repeated and in small quantities is needed to sustain the vital forces. Milk with lime-water, beef peptonoids, beef-juice, meat broths, Kumyss, egg albumen, water, etc., etc., may be given according to the exigencies of the case. If stimulation becomes necessary, alcohol in some form will be found useful, but should be given largely diluted in order not to disturb the digestion. Strychnia I believe to be our best respiratory heart and nerve stimulant and can always be given with benefit.

As said before, the liquefying and removal of tenacious and viscid exudate is always desirable. Ipecac (I prefer the wine) in minute doses, frequently repeated, will facilitate this process. The muriate, carbonate or aromatic spirits of ammonia are particularly useful and are general favorites. In cases where cyanosis and dyspnea are becoming urgent from accumulation of mucus in the bronchi, an emetic will oftentimes give great relief. Oxygen would also be indicated. In this connection I would like to quote a paragraph from a recent article by Dr. Chapin. He says: "As is well known, carbon-dioxide is produced more abundantly in early life than in later years, hence the lungs have relatively more work to perform. Not only are the lungs crippled in their natural work in pneumonia, but the high temperature and inflammatory action increase still more the production of carbon-dioxide, so that great strain is put upon the parts of these organs that are still able to perform their function. Add to this the filling of the bronchial tubes and the poor development of the respiratory muscles, and we can understand how a weakening and failure of respiration are what is constantly to be dreaded. The blood charged with carbon-dioxide tends to sluggish movement in the lungs, soon followed by exudation and emigration into the alveoli and tubes. The fatality would be much greater were it not for the compensation of a vigorous heart and blood vessels, and here is where the greatest advantage can be pursued in treatment. The right heart, which has to bear the brunt of the pulmonary obstruction, is relatively strong in early life, while the arteries, being healthy and elastic, aid the action of the left heart. . . . As the respiration becomes weaker and the lung more congested, the blood collects in the veins from the pulmonary obstruction, and the indication is to prevent stasis in the lungs by stimulating the heart, at the same time drawing off the blood as much as possible into the arteries. This may be accomplished by dilating the arteries, which, by lessening the amount of blood in the veins, takes off some of the pressure from the engorged pulmonary circulation. Nitro-glycerine is thus most valuable in relaxing the arterial system, and hence to a certain extent emptying the veins into the arteries."

This line of argument seems to me most rational, and I would like to know what the feeling of any of the members of the society is in regard to the use of nitro-glycerine in this condition. I personally have had no experience in the use of it in broncho-pneumonia, but shall feel decidedly like giving it a trial at the first opportunity where the indications seem to call for its use. On the other hand and for the opposite reason, namely, its tightening effect upon the arteries, digitalis is seldom used by the above author, or when employed is combined with a vaso-dilator.

It is not my purpose to enumerate all the drugs that have been recommended to meet the different indications during an attack of broncho-pneumonia; their number is legion. With a clear understanding of the conditions present and the dangers to be apprehended, a rational line of treatment will necessarily be followed. Avoid, however, the giving of many drugs, but rely principally upon good nursing, careful feeding, etc.

Just a word now in regard to the controlling of fever. When urgent symptoms are the result of high temperature much can be accomplished by the giving of a warm bath at 90° F , by the warm wet pack or by sponging. Ice bags to the head are a source of relief. The coal-tar derivatives are dangerous remedies in pneumonia and should never be used. Cold-compresses or ice-bags to the chest I am afraid of and think that if used at all should be accompanied with great precaution. The great majority of cases of broncho-pneumonia will require no treatment for fever. 'Tis only when dangerous heights are reached that active measures should be employed.

To briefly sum up then the treatment of broncho-pneumonia:

Support the vital forces.

Maintain the respiratory function.

Control hyperpyrexia.—*Southern California Practitioner.*

